# Acute Acalculous Cholecystitis with Empyema due to Salmonellosis

**DOI:** 10.1155/2019/5185314

**Published:** 2019-04-30

**Authors:** Georgios D. Lianos, Panagiota Drosou, Rizos Souvatzoglou, Anastasia Karampa, Georgios Vangelis, Emmanouil Angelakis, Vassilios Pappas, Epameinontas Lekkas

**Affiliations:** ^1^Department of Surgery, General Hospital of Preveza, Preveza, Greece; ^2^Anesthesiology Department, General Hospital of Preveza, Preveza, Greece

## Abstract

Empyema of acalculous gallbladder in the setting of salmonellosis represents an extremely rare and life-threatening clinical condition in adults. In this unique case report, we deal with a previously healthy patient who developed acalculous cholecystitis and empyema due to infection by* Salmonella*. He underwent explorative laparotomy in emergency setting, and cholecystectomy was performed due to his toxic clinical condition. Empyema of gallbladder was revealed and cultures were collected. A combination of antibiotics (ciprofloxacin and metronidazole) was set, and the patient was discharged 8 days after the surgical operation in good condition. It has to be highlighted that acalculous cholecystitis is a rare entity, mostly at critically ill patients, and treatment options depend on clinical condition, risk factors, and etiology. To our knowledge, this is the first case report dealing with acalculous cholecystitis with empyema due to salmonellosis up to date. Although it is extremely rare, high index of suspicion is needed by the operating surgeon in order to avoid unfavorable outcomes.

## 1. Introduction

Acalculous cholecystitis is an acute necroinflammatory disease of the gallbladder with a multifactorial pathogenesis. It accounts for approximately 5-10% of all cases of acute cholecystitis, is more fulminant than calculous cholecystitis, and is associated with high morbidity and mortality rates [1]. 

Pathogenetic factors are stasis, ischemia, cystic duct obstruction, and systemic illnesses, for example,* E. coli*,* Klebsiella pneumoniae*, and* Salmonella* species, which then cause inflammatory response in the gallbladder wall [2]. Acalculous cholecystitis is typically seen in critical illness, patients who are hospitalized with sepsis, burns and trauma, prolonged use of total parenteral nutrition, and older and immunosuppressed patients [3, 4].

Empyema of the gallbladder is one possible complication in the natural course of acute cholecystitis, it is a surgical emergency and demands a surgical intervention. To our knowledge, this is the first case report up to date dealing with empyema of the gallbladder due to salmonellosis in adults.

## 2. Case Report

A 32-year-old previously healthy male was admitted to our hospital with generalized abdominal pain, diarrhea, nausea, vomiting, and fever up to 39°. During assessment he was febrile without chills at 39°, BP at 135/70, pulse rate of 95 bpm, and respiratory rate at 20 breaths per minute. By his physical examination the abdomen was mildly tender to palpation with guarding in his epigastric and umbilical region.

Laboratory tests disclosed a white cell count of 14.4x1000/*μ*L with 92% neutrophils, 3.5% lymphocytes, normal red blood cell count (5x10^∧^6/*μ*L), haemoglobin (14.5g/dL), and platelets (160x1000/*μ*L). The biochemical studies including liver, renal, and coagulation profile were normal. There were a mild hyponatremia and hypokalemia and CRP was 8.56. Cultures were obtained from blood, stool samples were obtained, and ceftriaxone and metronidazole were empirically administered.

Furthermore, no abnormalities were detected on chest and abdomen X-ray whereas an urgent abdominal ultrasound revealed thickening of the gallbladder wall, gallbladder contraction, and a minor pericholecystic fluid collection, without dilation of common bile duct or intrahepatic biliary system. In view of the clinical and ultrasonographical findings, the patient was diagnosed with acute acalculous cholecystitis and he was hospitalized initially for fluids, antibiotics, and observation.

**Figure 1:**
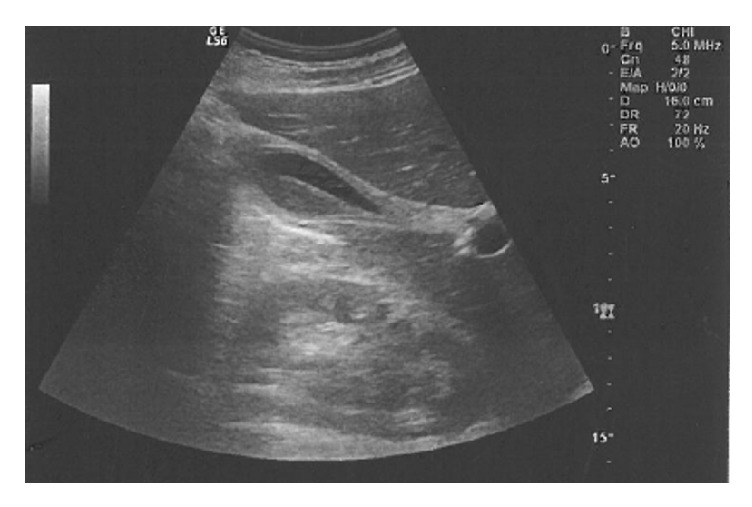
Abdominal ultrasound showing pericholecystic fluid collection, large free fluid at Douglas's pouch, and right paracolic gutter.

**Figure 2:**
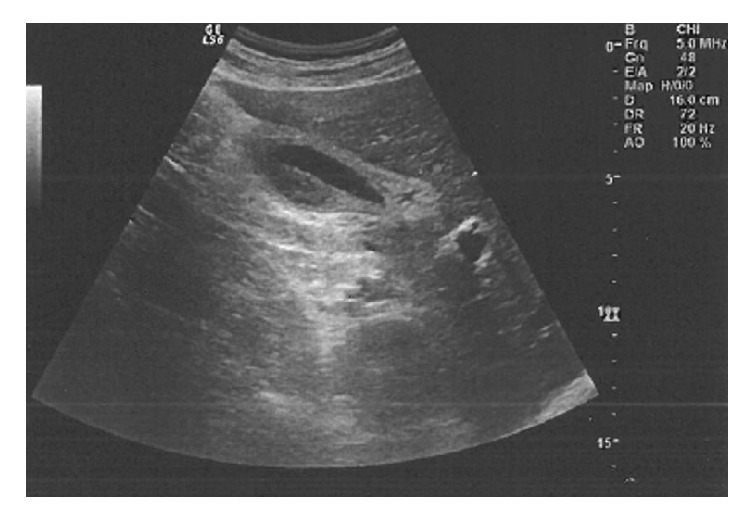
Abdominal ultrasound showing pericholecystic fluid collection, large free fluid at Douglas's pouch, and right paracolic gutter.

**Figure 3:**
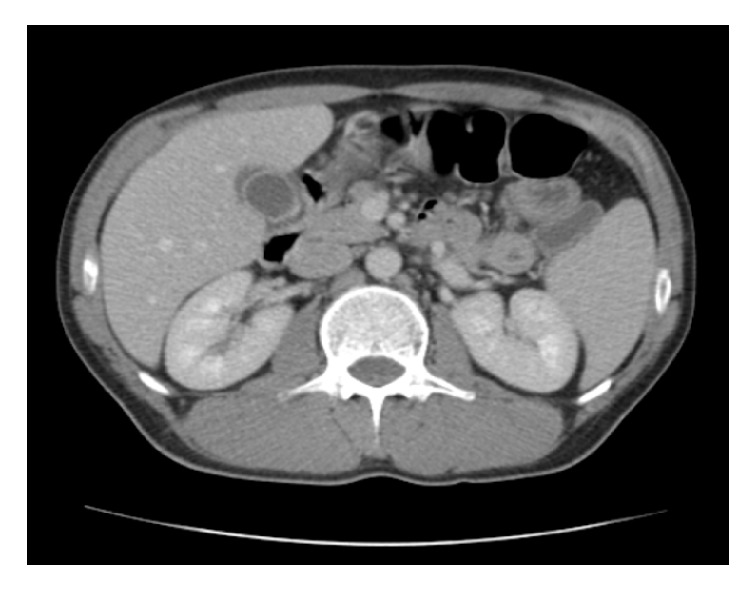
CT-scan of the abdomen showing thickening of the gallbladder wall and pericholecystic fluid collection.

**Figure 4:**
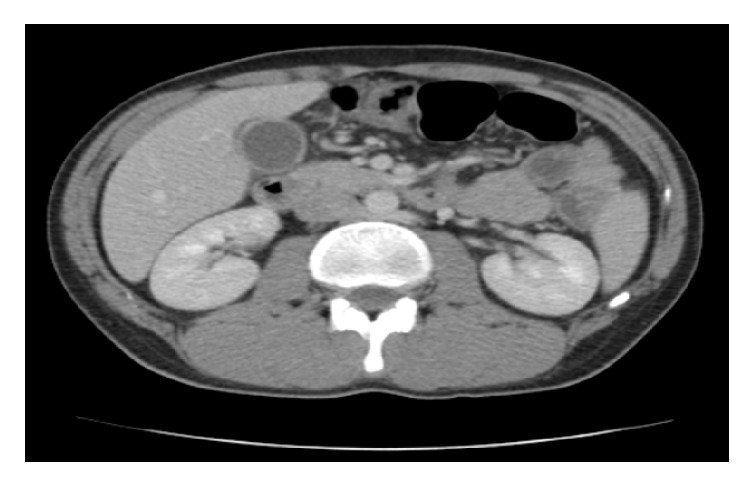
CT-scan of the abdomen showing thickening of the gallbladder wall and pericholecystic fluid collection.

After 36 hours of hospitalization, the patient was still febrile with fever up to 39°; however, there were obvious symptoms of toxicity. His vital points were 120bpm, BP 110/60, and 24 breaths/minute. By physical examination, the whole abdomen was contracted, with rebounding pain in the upper right upper quadrant region and Murphy's sign. Laboratory studies revealed precipitation of white blood cell count at 3.8x1000/*μ*L with 67.4% neutrophils and 18.9% lymphocytes. Red blood cells were at 4.2x10^∧^6/*μ*L, haemoglobin at 12.6g/dL, and platelets at 125.5x1000/*μ*L (Table 1). His abdominal ultrasound showed pericholecystic fluid collection, large free fluid at Douglas's pouch, and right paracolic gutter (Figures 1 and 2). The CT-scan of the abdomen showed thickening of the gallbladder wall, pericholecystic fluid collection, without biliary dilation, a large amount of free fluid at infrahepatic area and mostly in the pelvis, and right and left parabolic gutters (Figures 3 and 4).

The patient underwent exploratory laparotomy in emergency setting, and the findings were a swollen oedematous, acalculous gallbladder with empyema, and an amount of free fluid in the peritoneal cavity. After the check of the whole abdomen, he underwent cholecystectomy. Cultures were collected from the content of gallbladder and free fluid. At the same time the first cultures isolated* Salmonella* at stool and in the sequel; according to antimicrobial susceptibility test, he was given ciprofloxacin and metronidazole. Pathological findings confirmed the severity of cholecystitis. The culture from content of gallbladder isolated also* Salmonella*. He was discharged with oral antibiotics after 8 days of his admission.

## 3. Discussion

Salmonellae are motile gram-negative bacilli that colonize a wide range of mammalian hosts. They cause a broad range of infections, including gastroenteritis, enteric fever, bacteremia, and endovascular and focal infections such as abscesses. They can be divided into two broad categories: the typhoidal* Salmonella* (Typhi, Paratyphi) that causes typhoid and enteric fever and the much broader group of nontyphoidal* Salmonella* which causes gastroenteritis [5]. Symptoms of* Salmonella* gastroenteritis typically occur within 8 to 72 hours following ingestion usually of contaminated food or water, and the patient is presented with diarrhea, nausea, vomiting, fatigue, fever, chills, and abdominal pain [6, 7]. Fewer than 5% of documented* Salmonella*infection cases develop bacteremia, which can lead to a variety of extraintestinal manifestations such as endocarditis, myocarditis, pneumonia, pleural empyema, osteomyelitis, acalculous cholecystitis, hepatitis, hepatic abscesses, splenic abscess, peritonitis, paralytic ileus, and urinary tract infection [8, 9].

Lothrop, in 1915, first reported a case of acute acalculous cholecystitis as a complication of salmonellosis [10]. Nowadays, the pathophysiology is still incompletely defined. The mechanisms that have been proposed are a) the endotoxin-mediated reaction, seen in gram-negative sepsis, which leads to biliary stasis, increase of bile viscosity, sludge formation, and finally gallbladder mucosal damage; b) established bacteremia through portal vein directly to biliary system; c) the lymphatics drainage from gastrointestinal tract; and finally d) retrograde biliary carriage [11, 12].

The clinical manifestations of acute acalculous cholecystitis depend on the clinical status of the patient at the time of presentation. Fever, nausea, vomiting, diarrhea, abdominal pain, right upper quadrant pain, and leukocytosis are the initial symptoms, until the late complications such as sepsis and peritonitis [13]. When the acalculous cholecystitis is complicated by empyema of gallbladder, the patient becomes more toxic, with high spiking fever, chills, and finally septic shock. The diagnosis of acute acalculous cholecystitis as a complication of salmonellosis is based on clinical, laboratory, and radiological findings. In addition, the isolation of the pathogen in stool or blood cultures is also required.

Abdominal ultrasonography is considered the investigation of choice, and the criteria of acute acalculous cholecystitis include gallbladder wall thickness of >3mm, ultrasonographical Murphy's sigh, enlarged tense gallbladder, pericholecystic fluid, and absence of gall stones. Computed tomography and HIDA-scan are considered a good adjunct to ultrasound [4, 14].

Therapeutic management is controversial. Generally, if there are not any complications, such as empyema, gangrene, or perforation of the gallbladder, conservative treatment is sufficient. In case of complications the surgical treatment is mandatory. This can be performed with a simple percutaneous drainage, cholecystostomy (percutaneous or open), or cholecystectomy (laparoscopic or open) [14]. In our described case of grade II acute cholecystitis according to TOKYO guidelines [15], exploratory laparotomy was mandatory.

In summary, detention of acute acalculous cholecystitis, suspicion of its rare complications such as empyema, and early surgical intervention are crucial. The management of acalculous cholecystitis without complications is usually conservative, but when it is enchanting with empyema, gangrene, or perforation of gallbladder, the surgical treatment, as in our reported case, is absolutely necessary in order to avoid unfavorable outcomes.

## Figures and Tables

**Table 1:** Laboratory data at admission and after 36 hrs.

Laboratory Tests	Admission	After 36 hrs
white cell count	14.4x1000/*μ*L	3.8x1000/*μ*L
neutrophils	92%	67,4%
red blood cell count	5x10^∧^6/*μ*L	4.2x10^∧^6/*μ*L
haemoglobin	14.5g/dL	12.6g/dL
platelets	160x1000/*μ*L	125.5x1000/*μ*L

## References

[1] Iqbal S., Khajinoori M., Mooney B. (2018). A case report of acalculous cholecystitis due to Salmonella paratyphi B. *Radiology Case Reports*.

[2] Wang A.-J., Wang T.-E., Lin C.-C., Lin S.-C., Shih S.-C. (2003). Clinical predictors of severe gallbladder complications in acute acalculous cholecystitis. *World Journal of Gastroenterology*.

[3] Pelinka L. E., Schmidhammer R., Hamid L., Mauritz W., Redl H. (2003). Acute acalculous cholecystitis after trauma: a prospective study. *Journal of Trauma*.

[4] Ryu J. K., Ryu K. H., Kim K. H. (2003). Clinical features of acute acalculous cholecystitis. *Journal of Clinical Gastroenterology*.

[5] Acheson D., Hohmann E. L. (2001). Nontyphoidal salmonellosis. *Clinical Infectious Diseases*.

[6] Brooks J. T., Matyas B. T., Fontana J. (2012). An outbreak of Salmonella serotype typhimurium infections with an unusually long incubation period. *Foodborne Pathogens and Disease*.

[7] Mintz E. D., Cartter M. L., Hadler J. L., Wassell J. T., Zingeser J. A., Tauxe R. V. (1994). Dose–response effects in an outbreak of Salmonella enteritidis. *Epidemiology and Infection*.

[8] Saphra I., Winter J. W. (1957). Clinical manifestations of salmonellosis in man—an evaluation of 7,779 human infection identified at the New York Salmonella Center. *The New England Journal of Medicine*.

[9] Krueger A. L., Greene S. A., Barzilay E. J. (2014). Clinical outcomes of nalidixic acid, ceftriaxone, and multidrug-resistant nontyphoidal salmonella infections compared with pansusceptible infections in foodnet sites, 2006-2008. *Foodborne Pathogens and Disease*.

[10] Lothrop H. A. (1915). Acute cholecystitis complicating. *Annals of Surgery*.

[11] khan F. Y., Elouzi E. B., Asif M. (2009). Acute acalculous cholecystitis complicating typhoid fever in an adult patient: a case report and review of the literature. *Travel Medicine and Infectious Disease*.

[12] Bhutta Z. A. (2006). Current concepts in the diagnosis and treatment of typhoid fever. *British Medical Journal*.

[13] Savoca P. E., Longo W. E., Zucker K. A., McMillen M. M., Modlin I. M. (1990). The increasing prevalence of acalculous cholecystitis in outpatients: Results of a 7-year study. *Annals of Surgery*.

[14] Yasuda H., Takada T., Kawarada Y. (2007). Unusual cases of acute cholecystitis and cholangitis: Tokyo Guidelines. *Journal of Hepato-Biliary-Pancreatic Sciences*.

[15] Yokoe M., Takada T., Strasberg S. M. (2013). TG13 diagnostic criteria and severity grading of acute cholecystitis (with videos). *Journal of Hepato-Biliary-Pancreatic Sciences*.

